# Total Matrix Ca^2+^ Modulates Ca^2+^ Efflux via the Ca^2+^/H^+^ Exchanger in Cardiac Mitochondria

**DOI:** 10.3389/fphys.2020.510600

**Published:** 2020-09-16

**Authors:** Gayathri K. Natarajan, Lyall Glait, Jyotsna Mishra, David F. Stowe, Amadou K. S. Camara, Wai-Meng Kwok

**Affiliations:** ^1^Department of Anesthesiology, Medical College of Wisconsin, Milwaukee, WI, United States; ^2^Department of Physiology, Medical College of Wisconsin, Milwaukee, WI, United States; ^3^Cardiovascular Center, Medical College of Wisconsin, Milwaukee, WI, United States; ^4^Department of Biomedical Engineering, Medical College of Wisconsin and Marquette University, Milwaukee, WI, United States; ^5^Research Service, Veteran Affairs Medical Center, Milwaukee, WI, United States; ^6^Cancer Center, Medical College of Wisconsin, Milwaukee, WI, United States; ^7^Department of Pharmacology & Toxicology, Medical College of Wisconsin, Milwaukee, WI, United States

**Keywords:** CHE, Ca^2+^/H^+^ exchanger, LETM1, calcium retention capacity, total matrix Ca^2+^, cardiac mitochondria, pH gradient, Ca^2+^ efflux

## Abstract

Mitochondrial Ca^2+^ handling is accomplished by balancing Ca^2+^ uptake, primarily via the Ru360-sensitive mitochondrial calcium uniporter (MCU), Ca^2+^ buffering in the matrix and Ca^2+^ efflux mainly via Ca^2+^ ion exchangers, such as the Na^+^/Ca^2+^ exchanger (NCLX) and the Ca^2+^/H^+^ exchanger (CHE). The mechanism of CHE in cardiac mitochondria is not well-understood and its contribution to matrix Ca^2+^ regulation is thought to be negligible, despite higher expression of the putative CHE protein, LETM1, compared to hepatic mitochondria. In this study, Ca^2+^ efflux via the CHE was investigated in isolated rat cardiac mitochondria and permeabilized H9c2 cells. Mitochondria were exposed to (a) increasing matrix Ca^2+^ load via repetitive application of a finite CaCl_2_ bolus to the external medium and (b) change in the pH gradient across the inner mitochondrial membrane (IMM). Ca^2+^ efflux at different matrix Ca^2+^ loads was revealed by inhibiting Ca^2+^ uptake or reuptake with Ru360 after increasing number of CaCl_2_ boluses. In Na^+^-free experimental buffer and with Ca^2+^ uptake inhibited, the rate of Ca^2+^ efflux and steady-state free matrix Ca^2+^ [mCa^2+^]_ss_ increased as the number of administered CaCl_2_ boluses increased. ADP and cyclosporine A (CsA), which are known to increase Ca^2+^ buffering while maintaining a constant [mCa^2+^]_ss_, decreased the rate of Ca^2+^ efflux via the CHE, with a significantly greater decrease in the presence of ADP. ADP also increased Ca^2+^ buffering rate and decreased [mCa^2+^]_ss._ A change in the pH of the external medium to a more acidic value from 7.15 to 6.8∼6.9 caused a twofold increase in the Ca^2+^ efflux rate, while an alkaline change in pH from 7.15 to 7.4∼7.5 did not change the Ca^2+^ efflux rate. In addition, CHE activation was associated with membrane depolarization. Targeted transient knockdown of LETM1 in permeabilized H9c2 cells modulated Ca^2+^ efflux. The results indicate that Ca^2+^ efflux via the CHE in cardiac mitochondria is modulated by acidic buffer pH and by total matrix Ca^2+^. A mechanism is proposed whereby activation of CHE is sensitive to changes in both the matrix Ca^2+^ buffering system and the matrix free Ca^2+^ concentration.

## Introduction

Ca^2+^ handling in mitochondria is critical for cell survival. Recent research has focused on the role of mitochondria in dynamically regulating intracellular and steady-state free matrix Ca^2+^ ([mCa^2+^]_ss_) in response to increases in cytosolic Ca^2+^ (Duchen, 2000; Rizzuto et al., 2000; Csordás et al., 2012; Takeuchi et al., 2015; De Stefani et al., 2016). To achieve this, [mCa^2+^]_ss_ is tightly maintained through mechanisms that balance Ca^2+^ uptake from the cytosol, Ca^2+^ buffering in the matrix and Ca^2+^ efflux from the mitochondria (Finkel et al., 2015; Giorgi et al., 2018).

Ca^2+^ transport from the cytosol across the outer mitochondrial membrane (OMM) occurs mainly through the voltage-dependent anion channel (VDAC), which allows movement of a variety of ions, including both influx and efflux of Ca^2+^ (Tan and Colombini, 2007; Camara et al., 2017). Ca^2+^ uptake across the inner mitochondrial membrane (IMM) occurs mainly through the mitochondrial Ca^2+^ uniporter (MCU). MCU allows a selective influx of Ca^2+^ down its electrochemical gradient and is blocked by ruthenium red (RuR) and Ru360 (Kirichok et al., 2004; Gunter and Sheu, 2009; Baughman et al., 2011; De Stefani et al., 2011). The putative mitochondrial ryanodine receptor (mRyR) is also thought to contribute to Ca^2+^ influx in cardiac mitochondria (Beutner et al., 2005; Tewari et al., 2014), though its exact role remains unclear. mRyR is reported to be most active at lower concentrations of Ca^2+^ than those favored by MCU, and it is inhibited by RuR (Gunter and Sheu, 2009). Inside the mitochondrial matrix, Ca^2+^ is buffered primarily by precipitating with inorganic phosphate compounds, allowing for the uptake of large amounts of Ca^2+^ without disrupting the mitochondrial membrane potential, ΔΨ_*m*_ (Starkov, 2010; Wei et al., 2012).

Several exchangers are involved in Ca^2+^ transport across the IMM. The mitochondrial Na^+^/Ca^2+^ exchanger (NCLX) is well-characterized as the dominant Ca^2+^ efflux mechanism in excitable cells (Palty et al., 2010; Blomeyer et al., 2013, 2016; Boyman et al., 2013) and is powered by the Na^+^ electrochemical gradient across the IMM to allow Ca^2+^ efflux, although it can also act as a Li^+^/Ca^2+^ exchanger. The NCLX has been recently identified as a possible drug target for treating heart disease, specifically to protect against cardiac hypertrophy and sudden cardiac death (Luongo et al., 2017; Liu et al., 2018). The other putative Ca^2+^ efflux pathway, the Ca^2+^/H^+^ exchanger (CHE) is less studied and consequently, less understood. Early work indicated that a Na^+^-independent Ca^2+^ efflux (NICE) exists in both excitable and non-excitable tissues (Rizzuto et al., 1987). The contribution of this Ca^2+^ efflux mechanism, attributed to CHE, is suggested to be tissue-specific, with more activity in liver mitochondria (contributing up to 80% of total Ca^2+^ efflux), compared to cardiac mitochondria (<20% of total Ca^2+^ efflux) (Fiskum and Lehninger, 1979; Nicholls and Crompton, 1980; Rizzuto et al., 1987).

The recent discovery of the *Letm1* gene that is proposed to encode the CHE protein of the same name has provided some insights into the putative structure, function and mechanism of the NICE (Nowikovsky et al., 2012; Takeuchi et al., 2015; Austin and Nowikovsky, 2019; Li et al., 2019; Lin and Stathopulos, 2019). LETM1 (Leucine zipper-EF-hand containing transmembrane protein 1) was first described as a K^+^/H^+^ exchanger (KHE) in yeast mitochondria (Nowikovsky et al., 2004). Shortly after, a genome-wide RNAi screen of Drosophila predicted a Ca^2+^/H^+^ exchanger function for LETM1 (Jiang et al., 2009), which was reinforced by *in vitro* assays using isolated LETM1 reconstituted in liposomes (Tsai et al., 2014). Current evidence suggests that the LETM1 protein, in hexameric conformation, encodes for a CHE with electroneutral transport activity exchanging 1Ca^2+^ for 2H^+^ ions (Shao et al., 2016). However, questions regarding the primary function of LETM1 as a KHE or CHE are, as yet, unresolved and there is insufficient and conflicting evidence to confirm whether LETM1 as CHE contributes to Ca^2+^ uptake or Ca^2+^ efflux or is bidirectional (Ca^2+^ in/H^+^ out or H^+^ in/Ca^2+^ out) depending on the mitochondrial microenvironment (Jiang et al., 2013; Doonan et al., 2014; Tsai et al., 2014; Haumann et al., 2019).

The physiological significance of LETM1 as CHE was demonstrated in LETM1-deficient cell and animal models that exhibited impaired dynamics of Ca^2+^ uptake and efflux. For instance, LETM1 deletions have been linked to Wolf–Hirschhorn syndrome and heterozygous knockout mice show altered glucose regulation, reduced mitochondrial ATP production, and altered brain activity (Jiang et al., 2013). Furthermore, the potential involvement of LETM1 in K^+^ regulation and resulting regulation of mitochondrial Ca^2+^ may also have an impact on Na^+^ regulation and NCLX activity (Austin et al., 2017). LETM1 has been associated with delayed opening of the mitochondrial permeability transition pore (mPTP) in neurons lacking the PTEN-induced kinase 1 (PINK1), a condition that results in recessive familial Parkinson’s disease (Huang et al., 2017). However, such observations must be interpreted by taking into consideration that cation homeostasis may be altered and that any compensatory mechanisms may be unaccounted for. This leads to unanswered questions as to what regulates CHE activity in normal and dysfunctional mitochondria.

Previous studies have reported that CHE activity is more robust in hepatic mitochondria than in cardiac mitochondria, where the contribution of CHE to Ca^2+^ regulation is reportedly small (Crompton et al., 1977; Jurkowitz and Brierley, 1982; Gunter and Pfeiffer, 1990). Yet, expression of LETM1 protein in cardiac mitochondria is reported to be higher than in hepatic mitochondria (Uhlén et al., 2005; Fagerberg et al., 2014). In contrast to the accepted notion that CHE contributes little to Ca^2+^ regulation in cardiomyocytes, knockdown of LETM1 in rat neonatal cardiomyocytes decreased Ca^2+^ uptake as well as efflux (Doonan et al., 2014). Furthermore, a knockout of *Slc8b1*, the gene encoding NCLX in adult mice hearts decreased Ca^2+^ efflux by only 80% (Luongo et al., 2017), suggesting the presence and contribution of a significant Na^+^-independent efflux mechanism in adult ventricular cardiomyocytes. Thus, the expression density of LETM1 and its functional contribution to Ca^2+^ efflux as CHE appears to be discordant, suggesting that the factors affecting CHE activity are tissue-specific and hence warrant more investigation. Indeed, the higher expression of LETM1 in cardiac mitochondria suggests that, as CHE, it could be important in pH-sensitive conditions such as ischemia-reperfusion (IR) injury and provide a greater reserve capacity to modulate Ca^2+^ transport across the IMM.

To delineate the key modulators of CHE activity in cardiac mitochondria and the conditions under which CHE activity is enhanced, we (1) investigated the contribution of a NICE via CHE under basal conditions and (2) determined how changes in extra-matrix Ca^2+^ and/or pH modulate Ca^2+^ efflux via the CHE and intra-mitochondrial Ca^2+^ in isolated rat heart mitochondria. The results show that a basal NICE in cardiac mitochondria increases with extra-matrix acidification, indicating that H^+^ is a driving force for the exchanger. Furthermore, the rate of Ca^2+^ efflux was modulated by total matrix Ca^2+^ (mCa^2+^), indicating that Ca^2+^ efflux via CHE is modulated by both Ca^2+^ buffering and the [mCa^2+^]_ss_. In permeabilized H9c2 cells, the mitochondrial Ca^2+^ flux was modulated by targeted knockdown of LETM1. The results suggest that contribution of CHE in regulating matrix Ca^2+^ may be closely aligned with the Ca^2+^ retention capacity (CRC) of the mitochondria as well as changes in the extra-matrix pH.

## Materials and Methods

All animal use was approved by the Medical College of Wisconsin Institutional Animal Care and Use Committee (IACUC).

### Materials

All reagents and chemicals were purchased from Sigma-Aldrich (St. Louis, MO, United States) unless specified otherwise. Fluorescent dyes Fura-4F pentapotassium salt and Tetra Methyl Rhodamine (TMRM) were purchased from Life Technologies (Eugene, OR, United States). CGP37157 and Cyclosporine A (CsA) were purchased from Tocris Bioscience (Bristol, United Kingdom).

### Isolation of Mitochondria

Hearts were harvested from Sprague Dawley rats (male, ∼ 300 g wt), anesthetized intraperitoneally with Inactin and heparin and sacrificed. After mincing the tissue in ice-cold isolation buffer (200 mM mannitol, 50 mM sucrose, 5 mM KH_2_PO_4_, 5 mM MOPS, 1 mM EGTA, 5 U/ml of protease (*Bacillus licheniformis*), and 0.1% BSA, with pH adjusted to 7.15 with KOH), the suspension was homogenized for 15 s. After further addition of 17 ml isolation buffer, the suspension was again homogenized for 15 s. Mitochondria were isolated using differential centrifugation as described before (Haumann et al., 2010; Agarwal et al., 2012; Blomeyer et al., 2013, 2016; Mishra et al., 2019). Briefly, the homogenized tissue was first centrifuged at 8000 *g* for 10 min at 4°C. The pellet was resuspended in new isolation buffer and centrifuged at 850 *g* for 10 min. The resulting supernatant was then centrifuged at 8000 *g* for 10 min. The pellet from the third spin was resuspended in 0.1 ml of cold isolation buffer and stored on ice for further use. Protein concentration of the mitochondrial suspension was determined using a DU800 spectrophotometer (Bradford Method). Fresh isolation buffer was added to the mitochondrial suspension to obtain the final desired concentration of 12.5 mg protein/ml.

### Respiratory Control Index (RCI) Measurements

The quality of the isolated mitochondria was confirmed by measuring RCI to assess respiration using an oxygraph (Strathkelvin). Isolated mitochondria (0.5 mg/ml) added to experimental buffer (130 mM KCl, 5 mM K_2_HPO_4_, 20 mM MOPS, and 0.1% BSA, with pH adjusted to 7.15 with KOH) were energized with 10 mM freshly prepared K^+^-succinate (pH 7.15) substrate, followed by addition of 250 μM freshly prepared ADP. RCI was calculated by dividing the state 3 respiration slope (after adding ADP) by the state 4 respiration slope (after total consumption of ADP). The RCI of the mitochondria used in experiments ranged between 3 and 7. These values are consistent with reported RCI values for rat cardiac mitochondria energized with Complex II substrate (Hunter et al., 2012).

### Ca^2+^ Retention Capacity (CRC)

Extra-matrix Ca^2+^ was measured as described before (Mishra et al., 2019) by monitoring changes in the fluorescent Ca^2+^-sensitive dye Fura-4F pentapotassium salt (Excitation 340/380 nm, emission detection 510 nm) mixed with experimental buffer and using a cuvette-based spectrophotometer (Photon Technology International Inc.). Fura-4F was dissolved in DMSO. For all protocols, 960 μl of experimental buffer (130 mM KCl, 5 mM K_2_HPO_4_, 20 mM MOPS, and 0.1% BSA, with pH adjusted to 7.15 with KOH) containing Fura-4F (1 μM) was added to a cuvette. 40 μl of mitochondrial suspension was then added (0.5 mg/ml) to the experimental buffer to yield a final EGTA concentration of 40 μM in solution. At 1 min, mitochondria were energized with 10 μl of freshly prepared 1M K^+^-succinate (pH = 7.15). Succinate was used as the substrate, since the CRC of cardiac mitochondria has been found to be greater when respiring on complex II substrate compared to that when respiring on complex I substrate [unpublished observations from our group and (Madungwe et al., 2016; Matsuura et al., 2017)]. 20 μM pulses of CaCl_2_ were added to increase extra-matrix Ca^2+^, first at 3 min and every 5 min thereafter. The mPTP was considered open when the extra-matrix Ca^2+^ started to increase greatly over time, indicating a large net Ca^2+^ efflux.

To reveal Ca^2+^ efflux, Ca^2+^ uptake and reuptake through the MCU was blocked with 1 μM Ru360 and any Ca^2+^ influx through mRyR was blocked with 1 μM RuR (Blomeyer et al., 2016). Ru360 and RuR were added to the buffer solution 20 s after the application of a CaCl_2_ bolus. In some experiments, mitochondrial Ca^2+^ uptake was preserved by delaying opening of the mPTP with 500 nM CsA or 250 μM ADP. In other experiments (Figure 3C), the total matrix Ca^2+^ reserve was revealed by the application of 4 μM Carbonyl cyanide-4-(trifluorophenyl)-hydrazone (FCCP), which is a potent uncoupler of oxidative phosphorylation, leading to inhibition of ATP synthesis and membrane depolarization.

### Intra-Matrix Ca^2+^ Measurements

For measuring free matrix Ca^2+^, isolated mitochondria suspended in isolation buffer (5 mg/ml) were incubated with 5 μM Fura-4 AM for 20 min in dark at room temperature with stirring followed by centrifugation at 8000 g for 10 min at 4°C. The resulting mitochondrial pellet was re-suspended in fresh isolation buffer to a final concentration of 12.5 mg/ml and stored in ice. Aliquots (0.5 mg protein) were used in experiments using similar protocols as described for extra-matrix Ca^2+^ measurements.

### pH Assay

The effects of pH changes on the rate of Ca^2+^ efflux at steady-state was measured by addition of either HCl (final pH: 6.85 ± 0.05) or KOH (final pH: 7.45 ± 0.05) and monitoring changes in extra-matrix Ca^2+^. For all experiments in this series, mitochondria were energized with 10 mM K-succinate (at 60 s). CaCl_2_ boluses were added to the mitochondrial suspension at *t* = 180 s and *t* = 480 s and extra-matrix Ca^2+^ was allowed to reach a steady-state. The experiments then followed one of the following two protocols – in the first protocol, 1 μM Ru360 was added first (at 840 s), followed by either HCl or KOH (at 1200 s). In the second protocol, HCl or KOH was added first (at 840 s), followed by 1 μM Ru360.

### Membrane Potential (ΔΨ_m_) Measurements

Changes in ΔΨ_m_ were measured by ratiometric measurements of changes in the voltage-sensitive fluorescent dye, TMRM (Absorbance at 546 nm; emission at 573 nm) added to the experimental buffer to a final dye concentration of 1 μM.

Fluorescent Ratios (573 nm/546 nm) were measured and converted to estimated ΔΨ_m_ values against a calibration curve obtained using the method described in Scaduto and Grotyohann (1999). Briefly, fluorescence of TMRM dye (1 μM/ml of experimental buffer) was recorded before addition of mitochondria (0.5 mg/ml). K^+^-succinate (1 μM) and FCCP at concentrations (μM) 0, 0.25, 0.5, 1, 2, or 4 were added to the mitochondrial suspension. After 1 min, the fluorescence was recorded and the mixture was centrifuged at 8000 *g* for 5 min at 4°C to pellet the mitochondria. The concentration of TMRM remaining in the media ([TMRM]_O_) was calculated from a previously obtained fluorescence calibration assay by serial dilution of TMRM dye in experimental buffer. This value was subtracted from the initial amount of TMRM in the cuvette (before addition of mitochondria) to obtain the amount of TMRM associated with mitochondria ([TMRM]_*T*_). All concentration values were converted to a per mg basis. The concentration of free TMRM in the matrix ([TMRM]_*M*_) was calculated from the following equation:

$${\mathrm{[TMRM]}}_{T}=K_{i}*{\mathrm{(TMRM)}}_{M}+K_{O}*{\mathrm{(TMRM)}}_{O}$$

Units are: [TMRM]_*T*_ in (nmol/mg), (TMRM)_*M*_ and (TMRM)_*O*_ in (μl/mg), K_*i*_ and K_*o*_ are partition coefficients in (μl/mg).

ΔΨ_*m*_ was calculated using the Nernst equation

$$\Delta \psi_{m}=\frac{R*T}{z*F}ln\frac{{\left[TMRM\right]}_{O}}{{\left[TMRM\right]}_{M}}$$

The calculated ΔΨ_*m*_ was plotted against the fluorescent ratio measured before centrifugation to obtain the final calibration curve (Supplementary Figure S1). The calibration curve used in this study was:

$$\Delta \psi_{m}=-216.95*\frac{I_{573}}{I_{546}}+102.49$$

Values for K_*i*_ (0.5 μl/mg) and K_*o*_ (5 μl/mg) were estimated such that ΔΨ_*m*_ = ∼−180 mV in fully charged mitochondria when FCCP = 0 μM. The values of K_*o*_ and K_*i*_ were similar to the values of α (4.727) and ß (0.3799) obtained by Huang et al. (2007).

### Cell Culture and siRNA Transfection

H9c2 cells (CRL-1446, American Type Culture Collection, Gaithersburg, MD, United States) were cultured in Dulbecco’s modified Eagle’s medium (DMEM; Gibco, Carlsbad, CA, United States) supplemented with 10% (vol/vol) FBS and 5% 100 U/ml Penicillin-Streptomycin mixture at 37°C and 5% CO_2_. Seventy to eighty percent confluent cells were transfected with 15 nM of either universal negative control siRNA or a pool of 3 LETM1-targeted siRNA duplexes (Mission siRNAs, Sigma-Aldrich, St. Louis, MO, United States) using the Lipofectamine RNAiMAX transfection reagent (Invitrogen, Waltham, MA, United States) and used for experiments 48 h post-transfection. Rat LETM1 siRNA sense sequences were: GAGGAAAUGGCCCUGAAGA, CUGUAUCACGAGAUCCCUA and CGGAUUUGUGCAG ACCUCU.

### Validation of LETM1 Silencing by Quantitative Real-Time PCR Analysis

Total RNA was extracted from the siRNA-treated H9c2 cells using Trizol reagent (Invitrogen, Carlsbad, CA, United States). 1 μg of total RNA was converted to cDNA using random primers and SuperScript III First-Strand Synthesis System (Invitrogen, Carlsbad, CA, United States). mRNA levels of *LETM1* were measured by quantitative real-time PCR (qRT-PCR) using the SYBR Mix ExTaq (Applied Biosystems, Waltham, MA, United States). GAPDH was used as an endogenous control to normalize gene expression levels. The primers used for the analysis are: *Rat LETM1* 5′–ATC CCTACATCATTGCTCATACTG–3′ (forward) and 5′–CCTC CTTTGCCACAATTTCTG–3′ (reverse); *Rat GAPDH* 5′–ATG ACTCTACCCACGGCAAG –3′ (forward) and 5′–GGAA GATGGTGATGGGTTTC–3′ (reverse). Relative expression was calculated using the ΔΔC_*T*_ method.

### Protein Quantification by Western Blot

H9c2 cells were lysed in RIPA lysis buffer (Invitrogen, Carlsbad, CA, United States), supplemented with 1 mM PMSF and 1% protease inhibitor cocktail (Sigma-Aldrich). Lysates were incubated on ice for 30 min followed by centrifugation at 17,000 g for 15 min at 4°C. The supernatant was collected and quantified using the bicinchoninic acid assay (Thermo Fisher Scientific). Proteins were resolved on 4–20% mini-PROTEAN TGX gels (BioRad) and transferred to nitrocellulose membranes (0.45 μm, BioRad). The membranes were blotted with the following antibodies: Letm1 (16024-1-AP; Proteintech), NCLX (ab83551; Abcam), MCU (14997; Cell Signaling Technology) and GAPDH (CB1001; Millipore). GAPDH was used as the loading control. The LI-COR infrared fluorescent mouse (925–68020) and rabbit (925–32211) secondary antibodies were used for visualization by a LI-COR Odyssey scanner. Band quantification (densitometry) was performed using ImageJ and Origin 8 software (OriginLab Corporation, Northampton, MA, United States).

### Cell Permeabilization and Ca^2+^ Measurement Assay

Freshly dissociated wild-type (WT) or LETM1-siRNA treated (siLETM1) H9c2 cells were suspended in Ca^2+^-free Tyrode buffer (mM): 132 NaCl, 5 KCl, 10 HEPES, 5 Glucose, 1 MgCl_2_, pH = 7.4 (NaOH). For permeabilization, cells (1 × 10^6^ cells per experiment) were transferred to Buffer A (mM): 250 Sucrose, 100 HEPES, pH = 7.3 and incubated on ice for 10 min with 30 μg/ml digitonin (Sigma-Aldrich) in the presence of 20 μM EGTA and 1/200 EDTA-free protease inhibitor cocktail (Roche cOmplete via Sigma-Aldrich). The reaction was neutralized by addition of Buffer A and after centrifugation (500 g, RT, 5 min), the cells were resuspended in Na^+^-free experimental buffer. Experiments were performed in the presence of 1 μM Fura-4F penta potassium salt (to measure cytosolic Ca^2+^), 20 μM EGTA, 5 μM thapsigargin (to inhibit Ca^2+^ release from the sarcoplasmic reticulum) and 1M K^+^-Succinate (to energize complex II in mitochondria). As before, mitochondrial Ca^2+^ handling was observed by repetitive application of 5 μM CaCl_2_ boluses. Ca^2+^ efflux was revealed by the application of 1 μM Ru360 and the contribution of NCLX was eliminated by application of 2 μM CGP37157 (CGP) in the presence of Ru360.

### Analysis of Ca^2+^ Dynamics in WT and siLETM1 H9c2 Cells

Rate of change of [Ca^2+^]_*c*_ (d[Ca^2+^]_*c*_/dt) was analyzed for the 4th CaCl_2_ bolus for the traces shown in Figure 8D. For WT, d[Ca^2+^]_*c*_/dt was estimated as the slope “m” (in units of RFU/s) of the linear equation, y = m*x + c, used to fit the [Ca^2+^]_*c*_ response over 120 s following the application of the 4th CaCl_2_ bolus. For siLETM1, d[Ca^2+^]_*c*_/dt was estimated as the decay rate “τ” (in units of 1/s) of the single-exponential equation, y = y_0_ + a*exp(−x*τ), used to fit the [Ca^2+^]_*c*_ response over 120 s following the application of the 4th CaCl_2_ bolus.

### Protocols

The protocols for all experiments are described in their respective figure legends.

### Data Analysis

Data analysis was performed using Matlab 2016b (Mathworks, Natick, MA, United States). Efflux rate was calculated by fitting a linear regression to the data from 5–120 s after adding the agent of interest (Ru360, RuR, CsA, ADP, HCl or KOH). Buffering rates in the presence of Ru360, Ru360 + ADP and Ru360 + CsA were calculated by fitting single-exponentials to the individual raw traces in Origin 2017 (OriginLab Corporation). Statistics were run using Student’s *t*-test, with *p* < 0.05 considered significant.

## Results

### Na^+^-Independent Ca^2+^ Efflux in Cardiac Mitochondria Increases With Total Matrix Ca^2+^ Content

**FIGURE 1:**
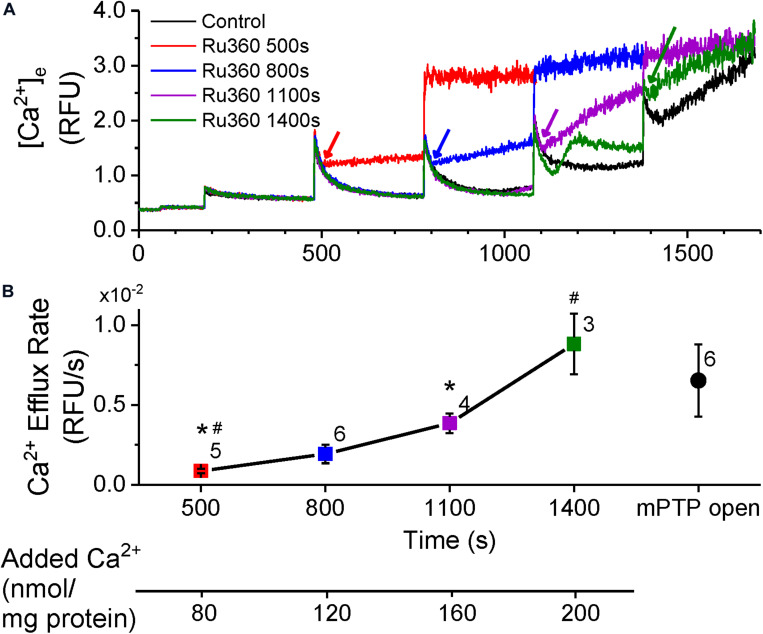
Ca^2+^-efflux increases with total matrix Ca^2+^. **(A)** Extra-matrix Ca^2+^ measured as relative fluorescence changes in Na^+^-free experimental buffer loaded with the Ca^2+^-sensitive ratiometric dye Fura-4F. 20 μM CaCl_2_ pulses were added first at 180 s and at every 300 s thereafter, in the absence (control, black trace) or the presence of MCU-inhibitor Ru360 (1 μM) added at different times as follows: red trace – 500 s; blue trace – 800 s; purple trace – 1110 s; green trace – 1400 s. Times of addition of Ru360 are indicated by color-matched arrows. **(B)** Efflux rates obtained from the slope values (m) calculated from fits (y = m*x + c) to the linear phase of Ca^2+^ efflux from 5 s to 1 min after addition of 1 μM Ru360 in each trace in **(A)**. Symbol colors correspond to the trace colors and conditions as specified in **(A)**. The efflux rate for Control (black circle) was obtained by fitting the equation (y = e^–(x–*t)/*τ^ + m*x + c) to changes in Ca^2+^ when mPTP opens (1380 s onward in control in **A**). Numbers besides symbols denote the number of experiments for each data point. The scale below the *X*-axis shows the cumulative Ca^2+^ added before the corresponding time-point of efflux rate calculation. *, # indicates significant difference (*p* < 0.05) between indicated pairs.

To monitor Ca^2+^ efflux with negligible contributions from NCLX, we exposed rat cardiac mitochondria, energized with K^+^-succinate, to bolus CaCl_2_ additions (20 μM) at 300 s intervals in Na^+^-free experimental buffer. Inhibition of Ca^2+^ reuptake with MCU-specific inhibitor, Ru360 (1 μM) just after a CaCl_2_ bolus revealed a net Ca^2+^ efflux, indicated by an increase in Ca^2+^ in the buffer solution (Figure 1A). Ru360 was applied at different time-points in the protocol, which correlated with the number of CaCl_2_ boluses added to the buffer. This allowed increasing amount of Ca^2+^ to be taken up and sequestered by the mitochondria, prior to revealing Ca^2+^ efflux. The Ca^2+^ efflux rate increased as the number of CaCl_2_ boluses increased, indicating that Ca^2+^ efflux was dependent on the amount of Ca^2+^ taken up by the mitochondria (Figure 1B). This is reflected in the cumulative amount of added Ca^2+^ at the time of Ru360 application (Figure 1B, lower panel). Interestingly, the Ca^2+^ efflux rate measured after Ru360 addition at 1400 s (following four CaCl_2_ boluses), approached the Ca^2+^ efflux rate observed upon opening of the mPTP at the same time in control (Figure 1A, black trace and Figure 1B, black circle). Since these experiments were conducted in Na^+^-free experimental conditions, the contribution of the NCLX or the Na^+^/H^+^ exchanger to the observed Ca^2+^ efflux can be ruled out. Thus, the results confirm the presence of a Na^+^-independent Ca^2+^ efflux that is revealed in the absence of Ca^2+^ reuptake via the MCU and in the absence of Ca^2+^ efflux via the NCLX. The results also show that the rate of the observed Ca^2+^ efflux is in direct proportion to the total matrix Ca^2+^. At very high total matrix Ca^2+^, near to the maximal (CRC) of the mitochondria, the NICE rate appeared to approach the rate of Ca^2+^ release observed during the irreversible opening of mPTP.

**FIGURE 2:**
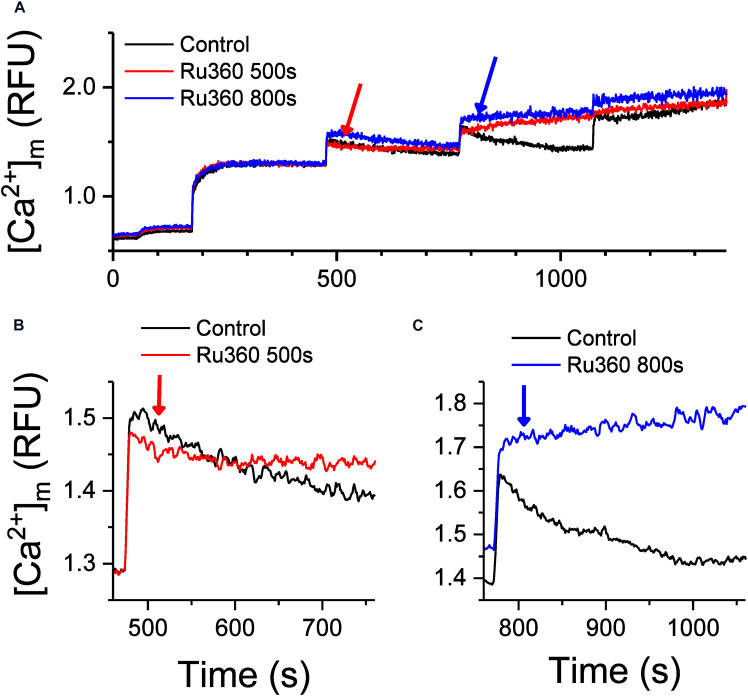
Inhibition of Ca^2+^ uptake regulates Ca^2+^ buffering and intra-matrix Ca^2+^. **(A)** Changes in intra-matrix Ca^2+^ in mitochondria isolated from rat hearts in response to 20 μM CaCl_2_ boluses in the absence (control, black) or presence of 1 μM Ru360 applied at 500 s (red) or 800 s (blue). **(B,C)** are expanded graphs of **(A)** to highlight the differences in the kinetics of Ca^2+^ buffering and steady state mCa^2+^ in response to application of Ru360.

Movement of Ca^2+^ from the cytosol and into the matrix is dictated by the membrane potential (ΔΨ_*m*_, ∼−180 mV, negative inside the matrix) across the IMM (Bernardi and Azzone, 1983) and by the free (unbound) Ca^2+^ concentration gradient between the intra- and extra-matrix compartments. In functioning mitochondria, ΔΨ_*m*_ and the Ca^2+^ gradient is maintained by regulating the free matrix Ca^2+^ concentration at a steady state (m[Ca^2+^]_ss_) via well-balanced mechanisms of Ca^2+^ uptake, efflux and buffering (Rizzuto et al., 2000; Nicholls, 2005; Finkel et al., 2015; Giorgi et al., 2018; Mishra et al., 2019). We recently showed that [mCa^2+^]_ss_ is maintained and returns to the same steady-state value between two bolus Ca^2+^ additions, thereby preserving ΔΨ_*m*_ and the Ca^2+^ gradient and promoting a robust Ca^2+^ uptake, until buffering fails and leads to mPTP opening (Mishra et al., 2019). Under our experimental control conditions at pH 7.15 (Figure 1A), the steady-state Ca^2+^ levels just prior to applying CaCl_2_ boluses at *t* = 480 s, *t* = 780 s and *t* = 1080 s were similar and the peak Ca^2+^ fluorescence after application of the CaCl_2_ boluses were also similar. This suggests that if, as previously reported, the [mCa^2+^]_ss_ is also similarly maintained between two successive CaCl_2_ pulses (Mishra et al., 2019), the Ca^2+^ efflux rate should also be maintained. Yet when Ca^2+^ uptake and any reuptake was inhibited with Ru360 at *t* = 500 s, *t* = 800 s and *t* = 1100 s respectively, the rate of Ca^2+^ efflux progressively increased from *t* = 500 to 800 s to 1100 s (Figure 1B). This suggests that the rate of Ca^2+^ efflux may be influenced by the amount of bound Ca^2+^ in the mitochondrial matrix. Hence, we measured changes in intra-matrix Ca^2+^ (Figure 2) using the same protocol as in Figure 1.

Addition of the first 20 μM CaCl_2_ bolus increased matrix Ca^2+^ to a new [mCa^2+^]_ss_. Subsequent boluses induced a sharp rise in [Ca^2+^]_*m*_ indicating rapid uptake via the MCU, which gradually decreases over time to [mCa^2+^]_ss_ indicative of Ca^2+^ buffering, until the CRC was exceeded leading to pore opening (Figure 2A, Control, black trace). Upon application of Ru360 at *t* = 500 s, Ca^2+^ continued to be buffered, but the eventual [mCa^2+^]_ss_ reached after 5 min was higher compared to control (Figure 2B, red versus black trace), indicating an altered equilibrium between buffered versus free [mCa^2+^]_ss_. Interestingly, when Ru360s was applied at *t* = 800 s, no more Ca^2+^ appeared to be buffered, instead there was a marked increase in [mCa^2+^]_ss_, suggesting a greater shift in the equilibrium between buffered versus [mCa^2+^]_ss_ in the absence of Ca^2+^ uptake (Figure 2C, blue versus black trace). This greater increase in [mCa^2+^]_ss_ also correlates with a greater rate of Ca^2+^ efflux observed in Figure 1 (blue trace). The effect of inhibiting Ca^2+^ uptake on [mCa^2+^]_ss_ is thus dependent on the time at which the inhibition occurs, which also correlates with an increase in total matrix Ca^2+^and the increasing rates of Ca^2+^ efflux observed at the same time points. The results thus far suggest that the rate of Ca^2+^ efflux in the absence of Ca^2+^ uptake is reflective of the total Ca^2+^ accumulated inside the matrix at any given time.

### ADP and CsA Decrease Ca^2+^ Efflux

**FIGURE 3:**
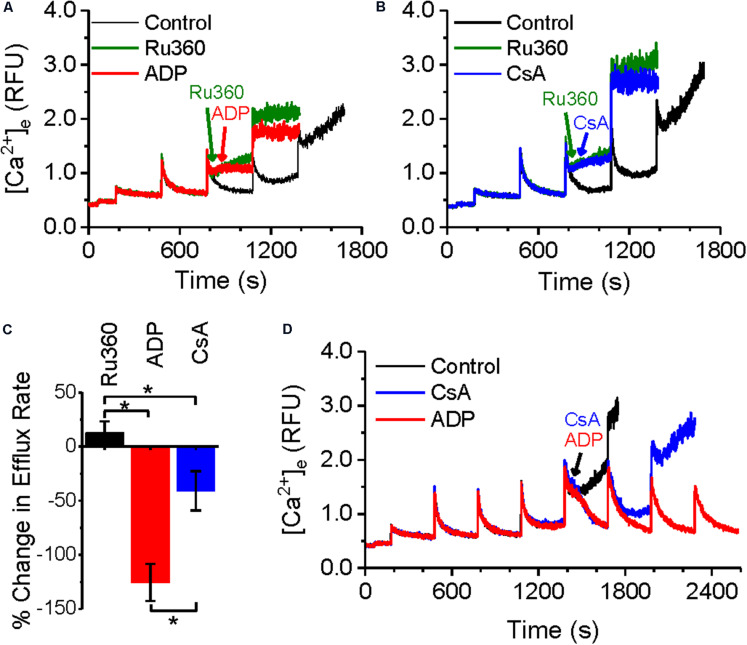
Effect of ADP and CsA on Ca^2+^-efflux and mPTP opening. **(A–C)** Representative raw traces of extra-matrix Ca^2+^ measured as relative fluorescence changes in Na^+^-free experimental buffer loaded with the Ca^2+^-sensitive ratiometric dye Fura-4F. 20 μM CaCl_2_ pulses were given at 180 and 480 s, followed by 1 μM Ru360 at 800 s (green trace), and **(A)** 250 μM ADP at 860 s or **(B)** 0.5 μM CsA at 860 s. **(C)** % change in the efflux rate before and after addition of 1 μM Ru360 (*n* = 9), 250 μM ADP (*n* = 6) or 0.5 μM CsA (*n* = 4). * indicates significant difference (*p* < 0.05) between indicated pairs. **(D)** Rescue of mPTP opening in Control (black trace) after addition of 250 μM ADP (red trace) or 0.5 μM CsA (blue trace).

We next tested whether agents that regulate Ca^2+^ buffering would affect the rate of Ca^2+^ efflux, using ADP and cyclosporine A (CsA), which have been reported to act as mitochondrial Ca^2+^ chelating/buffering agents. ADP is known to modulate [mCa^2+^]_ss_ by increasing Ca^2+^ buffering (Haumann et al., 2010) and in a recent study, CsA was shown to delay mPTP opening by promoting Ca^2+^ buffering mechanisms and maintaining low [mCa^2+^]_ss_ (Mishra et al., 2019). Both agents have been shown to also delay mPTP opening (Halestrap et al., 1997; Hausenloy et al., 2012; Sokolova et al., 2013; Haumann et al., 2019; Mishra et al., 2019). Figure 3 shows the effects of ADP and CsA on the rate of Ca^2+^ efflux as well as mPTP opening. As in Figure 1, application of Ru360 following a CaCl_2_ bolus addition revealed a Ca^2+^ efflux (Figure 3A, green trace). This efflux rate was significantly decreased by an application of 250 μM ADP, 20 s after the application of Ru360 (Figure 3A, red trace). A similar application of 500 nM CsA also decreased the rate of Ca^2+^ efflux (Figure 3B, blue trace) compared to that in the absence of CsA (Figure 3B, green trace). However, the decrease in Ca^2+^ efflux rate by CsA was significantly lower compared to that by ADP. The percent change in Ca^2+^ efflux rates are summarized in Figure 3C. Our results show that exogenous addition of Ca^2+^ chelators decreases the Ca^2+^ efflux rate from the mitochondria. Consistent with their effect on Ca^2+^ efflux rate, both ADP and CsA delayed mPTP opening, with ADP prolonging Ca^2+^ buffering and delaying mPTP opening much more than CsA (Figure 3D). Interestingly, prior to mPTP opening, both ADP and CsA, after application, returned the steady-state Ca^2+^ level to the same value (Figure 3D red and blue traces at *t* = 1680 s).

**FIGURE 4:**
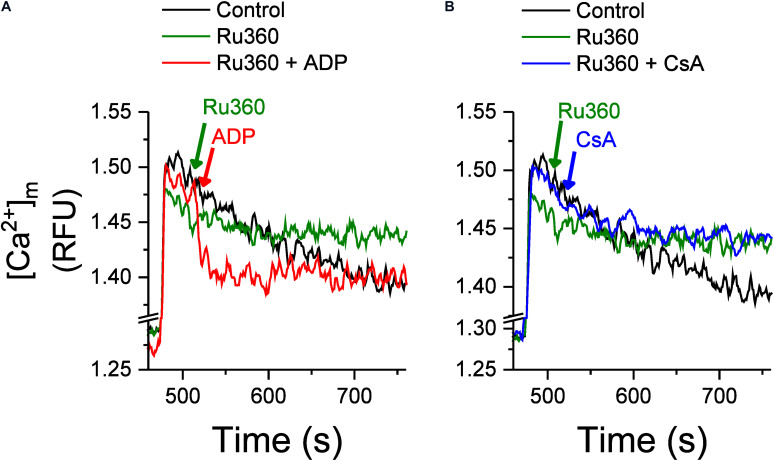
Effect of ADP and CsA on Ca^2+^ buffering and intra-matrix Ca^2+^. Changes in intra-matrix Ca^2+^ in mitochondria isolated from rat hearts in response to 20 μM CaCl_2_ bolus in the absence (control, black) or presence of 1 μM Ru360 applied at 500 s (green) followed by either **(A)** ADP (red) or **(B)** CsA (blue) at 520 s.

The effect of Ru360, ADP and CsA on intra-matrix Ca^2+^ (Figure 4) was consistent with their respective effect on the Ca^2+^ efflux rate (Figure 3). Addition of Ru360 alone resulted in a higher [mCa^2+^]_ss_ as before (Figure 2B). Following the addition of 250 μM ADP, 20 s after Ru360, there was a significant increase in the rate of Ca^2+^ buffering compared to Ru360 alone (rate of decay *τ* = 8.5 ± 0.3 s^–1^ in Ru360 + ADP versus *τ* = 18.3 ± 3.1 s^–1^ in Ru360 alone, *p* < 0.05, *n* = 3 for each condition), eventually reaching a lower [mCa^2+^]_ss_ than Ru360, but the same [mCa^2+^]_ss_ as in control (Figure 4A, red versus green and black traces). The faster rate of buffering and lower [mCa^2+^]_ss_ correlates with the significantly decreased rate of Ca^2+^ efflux observed in Figure 3, indicating that agents that chelate Ca^2+^ decrease the CHE-mediated Ca^2+^ efflux.

In contrast with the effect of ADP, the addition of 500 nM CsA, neither increased the rate of buffering (rate of decay τ = 21.1 ± 6.8 s^–1^ in Ru360 + CsA versus *τ* = 18.3 ± 3.1 s^–1^ in Ru360 alone, *n* = 3 for each condition) nor decreased the eventual [mCa^2+^]_ss_ compared to control or Ru360 alone (Figure 4B), even though the rate of Ca^2+^ efflux was significantly decreased compared to Ru360 alone (Figure 3C).

### Ca^2+^ Efflux Is Not Modulated by Mitochondrial RyRs or NCLX

**FIGURE 5:**
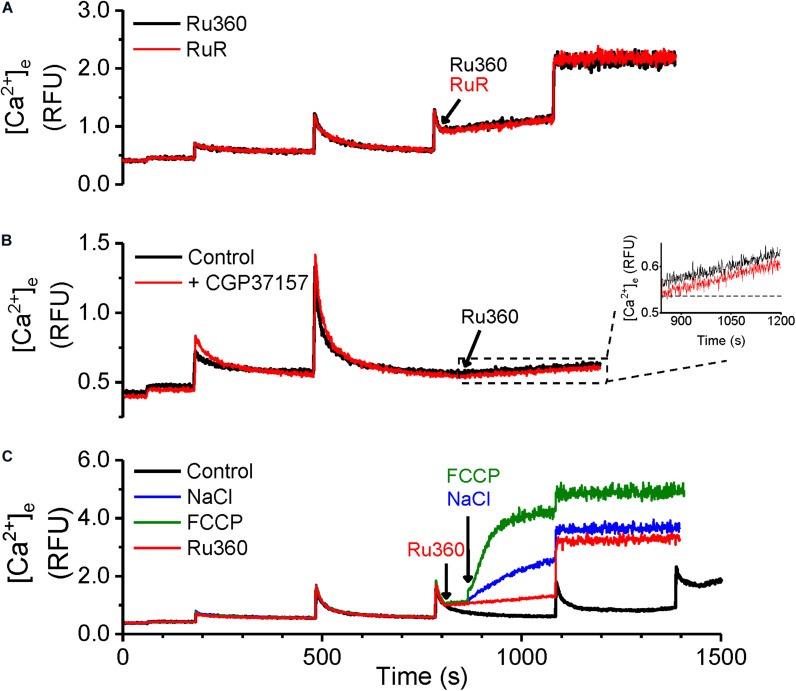
Ca^2+^-efflux is not modulated by mitochondrial RyRs or NCLX. **(A–C)** Representative raw traces of extra-matrix Ca^2+^ measured as relative fluorescence changes in Na^+^-free experimental buffer loaded with the Ca^2+^-sensitive ratiometric dye Fura-4F. **(A)** 20 μM CaCl_2_ pulses were given before and after addition of either 1 μM Ru360 (black trace) or 1 μM RuR, mRyR inhibitor Ruthenium Red (red trace). **(B)** 20 μM CaCl_2_ pulses were given at 180 and 480 s, followed by the addition of 1 μM Ru360 at 840 s in the absence (black trace) or the presence of NCLX inhibitor, CGP37157 (1 μM, red trace). **Inset:** Greater detail of traces enclosed by dashed box in **(B)** from *t* = 840 to 1200 s. **(C)** Control (black trace): 20 μM CaCl_2_ pulses were given at periodic intervals of 300 s until mPTP opening. Other traces: 20 μM CaCl_2_ pulses were given at 180, 480, and 780 s, followed by 1 μM Ru360 at 800 s (red trace), with further addition of 5 mM NaCl (blue trace), or 4 μM FCCP (green trace) at 820 s.

In Figures 1, 3 above, a net Ca^2+^ efflux, revealed by inhibition of Ca^2+^ reuptake via MCU, increased with an increase in matrix Ca^2+^. Besides the MCU, putative mitochondrial ryanodine receptors (RyRs) may be involved in Ca^2+^ uptake (Altschafl et al., 2007; Tewari et al., 2014) and hence may modulate the rate of Ca^2+^ efflux. To evaluate this possibility, we used the same protocol as in Figure 1 and recorded Ca^2+^ uptake and efflux in the presence of MCU-specific inhibitor Ru360 (Figure 5A, black trace) and in the presence of ruthenium red (RuR), which inhibits both the MCU and RyRs (Figure 5A, red trace). No differences were observed in the rate of Ca^2+^ efflux between the two protocols, indicating that Ca^2+^ uptake via mRyRs did not affect the observed rate of Ca^2+^ efflux.

Since our results were obtained in Na^+^-free experimental conditions, the contribution of the Na^2+^ gradient driven Ca^2+^ efflux via NCLX was obviated. However, it does not rule out a contribution of the NCLX operating in reverse mode to import Ca^2+^ into the matrix (Samanta et al., 2018) and modulate Ca^2+^ efflux when MCU-dependent Ca^2+^ uptake is inhibited with Ru360. However, no difference in Ca^2+^ efflux rate was observed when Ru360 was applied after attaining steady state following a bolus addition of CaCl_2_ in the absence (Figure 5B, black trace) or presence (Figure 5B, red trace) of CGP, a specific inhibitor of mitochondrial NCLX. The Ca^2+^ efflux rate in control and in the presence of CGP was 2.3 × 10^–4^ ± 5.1 × 10^–4^ RFU/s in control versus 2.4 × 10^–4^ ± 2.7 × 10^–5^ RFU/s in the presence of CGP (Figure 5B inset). These results confirm that, under our experimental conditions, the Ca^2+^ efflux rate was independent of modulation by mRyR and NCLX activity and could be solely attributed to the activity of the cardiac mitochondrial CHE.

In contrast with the Na^+^-free environment, we also compared the functionality of NCLX with that of CHE, by adding NaCl to the mitochondrial suspension. As shown in Figure 5C, addition of 5 mM NaCl following the addition of Ru360 substantially increased Ca^2+^ efflux (Figure 5C, blue trace), indicative of NCLX activity. Depolarization induced by addition of FCCP led to a robust Ca^2+^ efflux, as expected (Figure 5C, green trace). These experiments further corroborate the conclusion that the Ca^2+^ efflux observed in Figures 1, 3 solely reflect the activation of cardiac mitochondrial CHE and this activity is modulated by total cumulative matrix Ca^2+^.

### pH Changes Modulate Ca^2+^ Efflux

**FIGURE 6:**
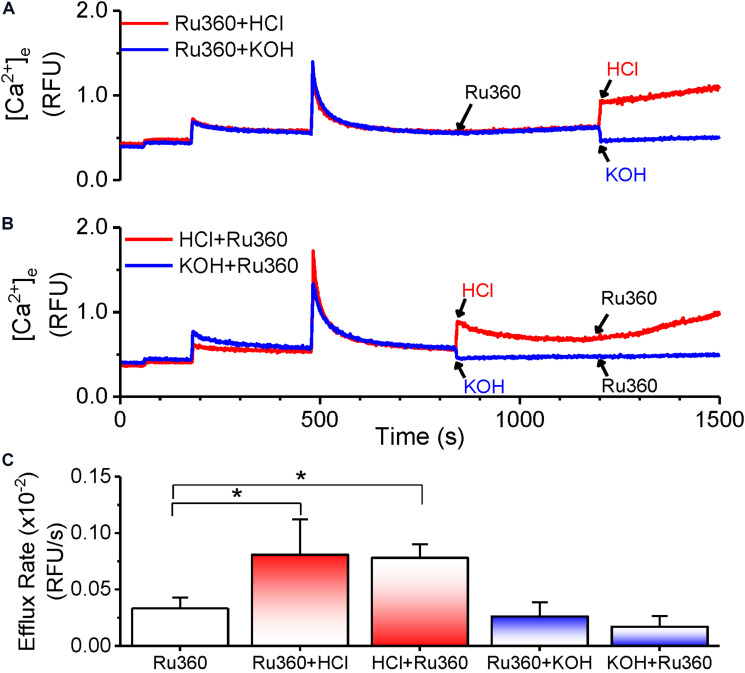
pH-dependent modulation of Ca^2+^-efflux rate. **(A,B)** Representative raw traces of extra-matrix Ca^2+^ measured as relative fluorescence intensity changes in Na^+^-free experimental buffer loaded with the Ca^2+^-sensitive ratiometric dye Fura-4F. 20 μM CaCl_2_ pulses were given at 180 and 480 s and allowed to reach steady state before applying different treatments as follows: **(A)** Addition of 1 μM Ru360 at 840 s followed by HCl (pH = 6.85 ± 0.05, red trace) or KOH (pH = 7.55 ± 0.05) at 1200 s **(B)** Addition of HCl (pH = 6.85 ± 0.05, red trace) or KOH (pH = 7.55 ± 0.05) at 840 s followed by 1 μM Ru360 at 1200 s. **(C)** Summary of efflux rates (m) calculated from fits (*y* = m*x + c) to the linear phase of Ca^2+^ efflux from 20 s to 1 min after the indicated intervention in **(A,B)**.

The CHE has been shown to function via the thermodynamically stable mechanism of driving Ca^2+^ transport using the H^+^ gradient in response to pH changes (Jiang et al., 2009; Tsai et al., 2014; Shao et al., 2016; Haumann et al., 2019). Our results thus far show that the Ca^2+^ efflux responds to changes in matrix Ca^2+^. We then investigated, whether the observed Ca^2+^ efflux is sensitive to pH changes. First, extra-matrix Ca^2+^ was allowed to reach a steady-state value following a bolus CaCl_2_ addition. This was followed by application of Ru360 to reveal a steady-state Ca^2+^ efflux (Figure 6A). Subsequently, a change in the pH of the external solution from the basal 7.15 ± 0.03 to a more acidic value of 6.85 ± 0.05 was induced by the addition of HCl. This resulted in an initial instantaneous jump in Ca^2+^ fluorescence, reflecting the aggregate of a decreased affinity of EGTA as well as the dye for Ca^2+^ in acidic pH (Lattanzio, 1990), followed by the continuation of the Ca^2+^ efflux at a higher rate, indicated by the increase in the slope of the Ca^2+^ efflux signal compared to the slope observed before addition of HCl (Figure 6A, red trace). The calculated rate of Ca^2+^ efflux was 2.4-fold greater after HCl addition (Figure 6C, Ru360 + HCl) compared to before (Figure 6C, Ru360 alone). When the pH was changed from the basal 7.15 ± 0.03 to a more alkaline pH 7.55 ± 0.05 by addition of KOH, there was an initial instantaneous decrease in fluorescence consistent with an increased affinity of EGTA and the dye to Ca^2+^. However, in contrast with the observation in acidic buffer pH, no further change in the rate of Ca^2+^ efflux was observed in alkaline buffer pH (Figure 6A, blue trace and Figure 6C, Ru360 + KOH).

We next reversed the order of application of HCl and Ru360 to first activate the CHE before inhibiting MCU-driven Ca^2+^ uptake. The rate of Ca^2+^ efflux observed upon addition of Ru360 was greater than that in Ru360 alone and not significantly different from the rate of Ca^2+^ efflux observed when HCl was applied after Ru360 (Figure 6B, red trace and Figure 6C, HCl + Ru360). Consistent with the effect observed in Figure 6A, reverse application of KOH first, followed by Ru360 did not change the rate of Ca^2+^ efflux (Figure 6B, blue trace and Figure 6C, KOH + Ru360).

The results here indicate that changes in extra-matrix pH, specifically changes that make it more acidic are able to modulate Ca^2+^ efflux and further confirm that the observed Ca^2+^ efflux is due to the activation of CHE by the proton gradient.

### CHE Activity Correlates With Changes in Membrane Potential

**FIGURE 7:**
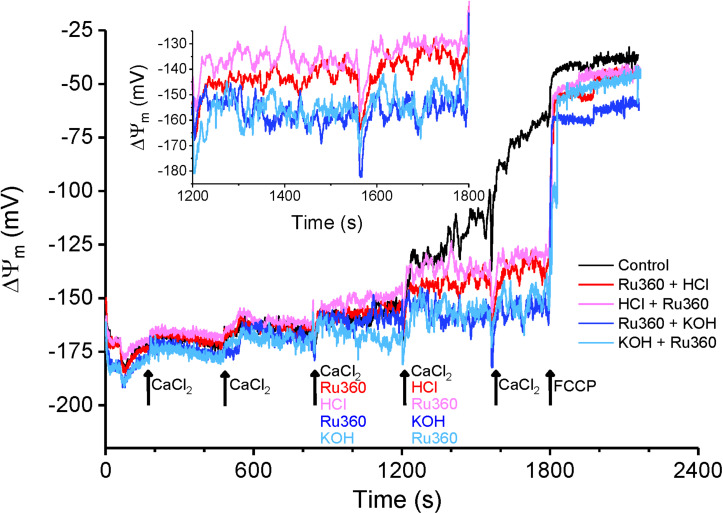
Membrane potential changes in response to acidic and basic pH. Average ΔΨ_*m*_ calculated as a function of TMRM intensity changes as described in section “Materials and Methods.” Membrane potential was evaluated for Control (black trace) and for interventions as indicated by arrows. For all traces, maximal depolarization was observed with the addition of 1 μM FCCP at t = 1800 s. **Inset:** Enhanced detail of the graph between 1200 and 1800 s shows a depolarization caused by a change to more acidic pH in the buffer solution.

According to the chemiosmotic principle, Ca^2+^ uptake into the mitochondria is primarily regulated by the large ΔΨ_*m*_, generated by the pumping of H^+^ ions by the electron transport chain. In contrast, Ca^2+^ efflux, initially characterized in liver mitochondria, was determined to be independent of ΔΨ_*m*_ (Pozzan et al., 1977; Gunter et al., 1983; Brand, 1985) and instead determined by the chemical gradient of the ions being transported across the IMM. The consensus opinion is that, in normal conditions, Ca^2+^ efflux matches Ca^2+^ influx to maintain a steady state [Ca^2+^]_*m*_ and ΔΨ_*m*_. In our study, application of Ru360 inhibited Ca^2+^ reuptake and revealed a Ca^2+^ efflux (Figures 1, 3, 5, 6), but caused little change in ΔΨ_*m*_ (Figure 7, red and blue traces at 840 s). This suggests that under basal conditions, Ca^2+^ efflux via the CHE does not directly impact ΔΨ_*m*_. Repetitive bolus CaCl_2_ additions eventually depolarized ΔΨ_*m*_ (Figure 7, Control, black trace), which correlates to the times at which matrix CRC reached threshold for mPTP opening under similar experimental conditions (Figure 1, Control, black trace, *t* ≥ 1100 s). The observed depolarization also correlated with the times at which the rate of Ca^2+^ efflux via the CHE was increased as revealed in the presence of Ru360 (Figure 1B).

In contrast, an extra-matrix pH change to a more acidic value in the presence of Ru360 (Figure 7, red trace at 1200 s) and application of Ru360 after a change to a more acidic buffer pH (Figure 7, pink trace at 1200 s) caused a small depolarization of ΔΨ_*m*_ = ∼20 mV (Figure 7 inset). Under these conditions, the mitochondria attained a new steady-state ΔΨ_*m*_ and this correlates with an increased rate of Ca^2+^ efflux observed in similar experimental conditions (Figure 6A, red trace and Figure 6B, red trace and Figure 6C, Ru360 + HCl and HCl + Ru360). The observation that in acidic buffer pH, ΔΨ_*m*_ is depolarized and the rate of Ca^2+^ efflux is increased is consistent with the activation of CHE due to an increase in the H^+^ gradient, as also reported by Haumann et al. (2019). It is interesting that acidic buffer pH increases Ca^2+^efflux rate and is associated with depolarization, while mCa^2+^-driven increase in Ca^2+^ efflux rate at physiological pH is associated with depolarization after the mitochondria reaches its maximum buffering capacity (Figure 7, Control, black trace). Taken together, our results show that changes in extra-matrix pH as well as increases in matrix Ca^2+^ modulate Ca^2+^ efflux via the CHE and both are associated with a depolarization of ΔΨ_*m*_.

### Knockdown of LETM1 Modulates Mitochondrial Ca^2+^ Efflux

**FIGURE 8:**
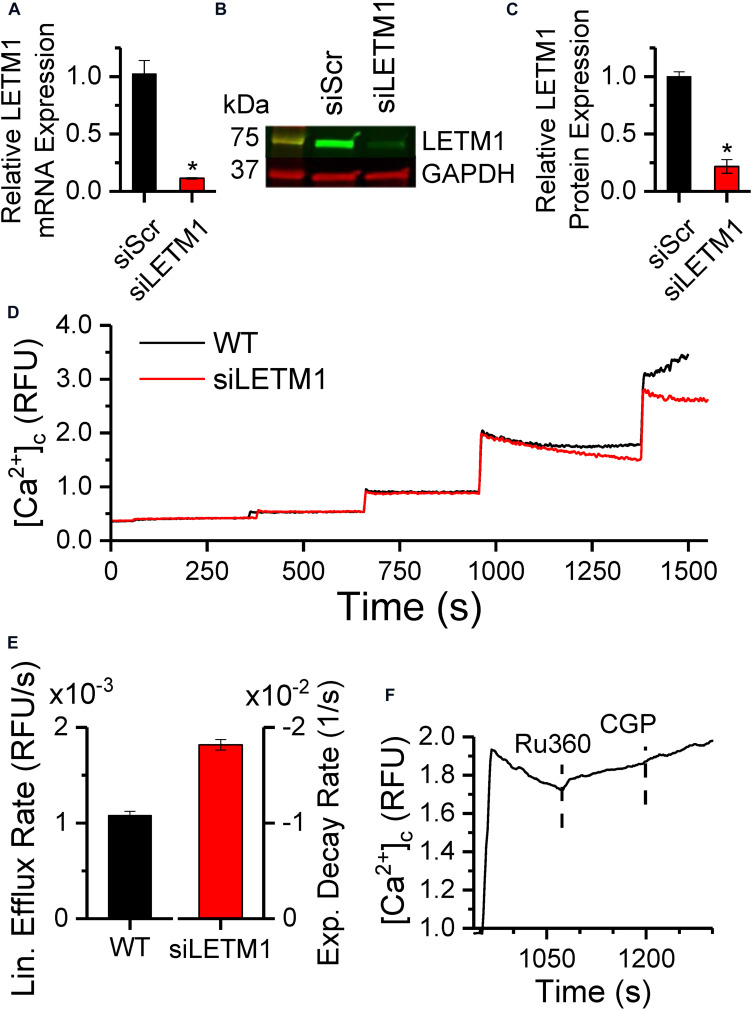
LETM1 knockdown modulates Ca^2+^ efflux in permeabilized H9c2 cells. **(A)** mRNA expression of LETM1 in H9c2 cells transiently transfected with scrambled (siScr) or LETM1 siRNA (siLETM1) for 48 h, normalized to the GAPDH mRNA level. **(B)** Representative immunoblot and **(C)** densitometric quantification of LETM1 protein expression in H9c2 cells treated with scrambled or LETM1 siRNA for 48 h, with GAPDH as loading control. Bars in **(A,C)** represent mean ± SEM (*n* = 3) relative to scrambled siRNA. *p < 0.0005 versus. control siRNA. **(D)** Changes in cytosolic Ca^2+^ in permeabilized WT H9c2 cells (black) or H9c2 cells transfected with LETM1 siRNAs (red), in response to 5 μM CaCl_2_ boluses when energized with 10 mM K-Succinate. **(E)** Rate of change of Ca^2+^ calculated using linear fits for Ca^2+^ efflux rate in WT and 1-exponential fits for the decay rate in [Ca^2+^]_*c*_ in siLETM1 H9c2 cells following the 4th CaCl_2_ bolus in **(D)**. Further details provided in section “Materials and Methods.” **(F)** Changes in cytosolic Ca^2+^ in permeabilized WT H9c2 cells in response to a 5 μM CaCl_2_ bolus followed by addition of 1 μM Ru360 and 2 μM CGP at the times indicated by dashed lines.

To verify whether the mCa^2+^ and H^+^-driven Ca^2+^ efflux is mediated by the putative CHE protein LETM1, we performed a transient targeted knockdown of LETM1 in H9c2 cardiac myoblasts (Figure 8). Transfection of H9c2 cells with a pool of siRNAs targeting LETM1 led to significant downregulation of LETM1 mRNA levels (0.12 ± 0.006-fold, Figure 8A) and protein expression (0.22 ± 0.005-fold, Figures 8B,C). No significant changes were observed in the expression of MCU and NCLX proteins (Supplementary Figure S2). Ca^2+^ dynamics were measured in permeabilized wild-type (WT) or LETM1 siRNA-treated (siLETM1) H9c2 cells energized with 10 mM K-Suc (Figure 8D). To eliminate ER/SR-mediated Ca^2+^ fluxes, 5 μM thapsigargin and 20 μM EGTA were added to the Na^+^-free experimental buffer. Upon repetitive application of 5 μM CaCl_2_ boluses, WT and siLETM1 H9c2 cells exhibited similar Ca^2+^ dynamics for the first three pulses. However, application of a fourth CaCl_2_ bolus induced a linear Ca^2+^-efflux in WT cells that was eliminated in siLETM1 H9c2 cells, indicating the presence of a LETM1-mediated Ca^2+^ efflux in the WT cells (Figure 8D). The rate of Ca^2+^ efflux in WT H9c2 cells was 0.11 × 10^–2^ ± 4.39e-5 rfu/s. In contrast the Ca^2+^ dynamics for the 4th CaCl_2_ bolus in siLETM1 H9c2 cells exhibited an exponential decay with a rate constant of 0.018 ± 5.5e-4 sec^–1^ (Figure 8E). Since these studies were performed in Na^+^-free medium and inhibition with CGP did not change the Ru360-induced Ca^2+^ efflux rate in WT cells (Figure 8F), we conclude that any Ca^2+^ efflux mediated by NCLX was suppressed under our recording conditions.

## Discussion

The novel findings of this study are that in cardiac mitochondria, inhibition of MCU-mediated Ca^2+^ uptake (1) reveals a Ca^2+^ efflux that is modulated by changes in matrix free Ca^2+^, Ca^2+^ buffering and pH, (2) increases the rate of Ca^2+^ efflux as well as free matrix Ca^2+^. (3) CHE activity is associated with depolarization of the mitochondrial membrane potential. (4) Ca^2+^ dynamics in H9c2 cells is modified in the absence of LETM1.

Earlier studies stipulated that Ca^2+^ uptake was balanced by Ca^2+^ efflux mechanisms to maintain [mCa^2+^]_ss_, since inhibition of MCU-dependent Ca^2+^ uptake by ruthenium red (RuR) or Ru360 had no effect on ΔΨ_*m*_ (De Stefani et al., 2016). Later studies showed that Ca^2+^ efflux via CHE occurs in a ΔΨ_*m*_-independent manner and is instead powered by a ΔpH gradient across the IMM (Brand, 1985; Jiang et al., 2009; Tsai et al., 2014; Shao et al., 2016; Haumann et al., 2019). This study demonstrates a novel mechanism for the modulation of CHE-mediated Ca^2+^ efflux by Ca^2+^ buffering.

An intriguing observation is that Ru360 increased Ca^2+^ efflux as well as free matrix Ca^2+^ (Figure 2A) and this effect was enhanced at greater matrix Ca^2+^ loads (Figure 2B). With Ca^2+^ uptake inhibited and with an active Ca^2+^ efflux, it is expected that free matrix Ca^2+^ would decrease. Instead, whether due to a direct effect of Ru360 or due to a shifting equilibrium between Ca^2+^ uptake, efflux and buffering, the result is an increase in free matrix Ca^2+^ due to an apparent reduction in Ca^2+^ buffering. This notion is further bolstered by the effect of ADP, which increased the rate of Ca^2+^ buffering, leading to a decrease in free matrix Ca^2+^ and a decrease in the availability of Ca^2+^ to bind and activate the CHE. Together with the observations of extra-matrix Ca^2+^ changes (Figure 1), the results of our study suggest that Ca^2+^ efflux rate via CHE is determined by more than just a Ca^2+^ or pH gradient and is modulated by Ca^2+^ buffering as well.

CsA decreased the rate of Ca^2+^ efflux, but to a lesser extent than ADP and without decreasing free matrix Ca^2+^ or increasing Ca^2+^ buffering. Although the underlying mechanism is unclear, that CsA decreased the Ca^2+^ efflux rate via diminished flickering of the mPTP pore cannot be excluded.

The CHE is encoded by the mitochondrial protein LETM1 (Jiang et al., 2009; Doonan et al., 2014; Tsai et al., 2014; Shao et al., 2016), although it was first identified as a KHE responsible for mitochondrial dysmorphism in patients with the Wolf-Hirschhorn syndrome (Nowikovsky et al., 2004). Questions exist regarding the assembly and stoichiometry of CHE formation and the mechanism by which LETM1 regulates mitochondrial Ca^2+^ and/or K^+^ flux. However, *in vitro* systems have established that LETM1 includes one or more EF-hands in its sequence and conducts its exchanger activity via the thermodynamically stable mechanism of driving Ca^2+^ transport using the H^+^ gradient in response to pH changes. Our study in isolated mitochondria suggests that in addition to changes in pH (Figure 6), the CHE is also modulated by total matrix Ca^2+^ (Figures 1–4). These observations receive support from whole-cell studies, where a transient knockdown of the putative CHE protein, LETM1, changed the dynamics of Ca^2+^ efflux in permeabilized H9c2 cells (Figure 8).

**FIGURE 9:**
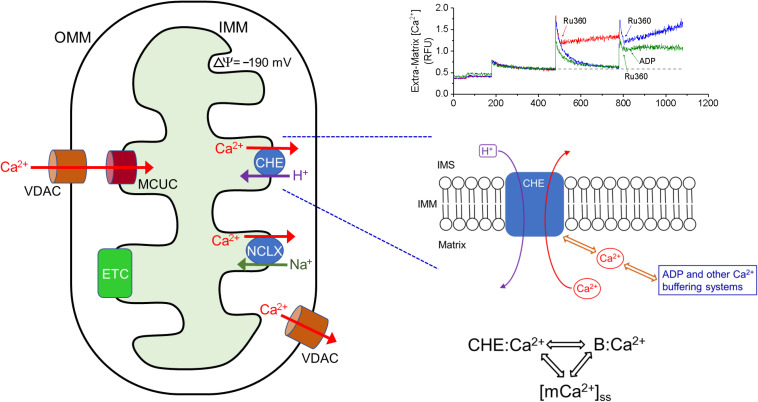
Ca^2+^ efflux via CHE is sensitive to total matrix Ca^2+^. **Left:** Representation of the principal influx and efflux pathways of mitochondrial Ca^2+^. OMM: Outer Mitochondrial Membrane, VDAC: Voltage-Dependent Anion Channel, IMM: Inner Mitochondrial Membrane, ETC: Electron Transport Chain, MCUC: Mitochondrial Calcium Uniporter Complex, NCLX: Na^+^-Ca^2+^-Li^3+^-Exchanger, CHE: Ca^2+^/H^+^ Exchanger. **Bottom Right:** Proposed scheme for the activation of CHE by total matrix Ca^2+^. Ca^2+^ efflux rate via CHE activation may be modulated by both the free steady-state matrix Ca^2+^ and by the amount of Ca^2+^ bound to other matrix buffering agents. By this scheme, the rate of Ca^2+^ efflux via CHE will increase as steady state matrix Ca^2+^ increases or when buffering capacity is exceeded. CHE:Ca^2+^ denotes Ca^2+^ bound to the CHE protein, B:Ca^2+^ denotes Ca^2+^ bound to matrix buffering agents such as ADP, [mCa^2+^]_ss_ denotes steady-state matrix free Ca^2+^ concentration. **Top Right:** Superimposed traces of extra-matrix Ca^2+^, adapted from Figure 1A, showing the different rates of Ca^2+^ efflux, when Ru360 is applied after addition of one Ca^2+^ bolus (red) or two CaCl_2_ boluses (blue) or two CaCl_2_ boluses and addition of ADP 20 s after Ru360 (green). In all three cases, the starting baseline Ca^2+^ was the same and the applied CaCl_2_ bolus was the same.

Our observations lead us to propose a novel three-way dynamic relationship between CHE activation, Ca^2+^ buffering and matrix Ca^2+^ whereby the activation of CHE depends on how much Ca^2+^ is accessible to bind to the CHE protein, either through changes in matrix free Ca^2+^ or buffered Ca^2+^ (Figure 9). According to this scheme, on the one hand, Ca^2+^ efflux via the CHE would increase under conditions where Ca^2+^ buffering decreases/fails or matrix steady-state free Ca^2+^ increases (for instance, due to increased Ca^2+^ uptake or reduced Ca^2+^ efflux via NCLX), leading to more Ca^2+^ to bind to CHE. On the other hand, processes that increase Ca^2+^ buffering or decrease the matrix free Ca^2+^ (through reduced Ca^2+^ uptake or greater efflux via NCLX) would decrease Ca^2+^ efflux via the CHE by making less Ca^2+^ accessible to bind to CHE. Such a scheme would explain the effect of ADP on the Ca^2+^ efflux rate. Under conditions where matrix Ca^2+^ is maintained at a steady state, if ADP sequesters more Ca^2+^, less free Ca^2+^ would be available to bind to CHE, leading to decreased activity of the exchanger and subsequently, reduced Ca^2+^ efflux. The reverse would be true when, at near buffering capacity, less Ca^2+^ would be buffered and more Ca^2+^ would be available to bind to CHE and increase the rate of Ca^2+^ efflux. Additional experiments would be needed to confirm this notion.

It is also to be noted that in our study, which uses mitochondria from rat heart, ADP is more effective in delaying mPTP opening, whereas Mishra et al. (2019) reported a significantly greater effect of CsA in delaying mPTP opening compared to ADP in mitochondria from guinea pig heart. The species-specific modulation of Ca^2+^ buffering and delay in mPTP opening by CsA and ADP suggests that, the mechanisms that regulate CHE activity will also be similarly impacted by tissue-specific and species-specific differences.

Acidic buffer pH (between 6.8 and 6.9 pH units) depolarized the IMM and increased the rate of Ca^2+^ efflux (Figures 6, 7). In experiments, where HCl was added before Ru360, a small increase in Ca^2+^ was evoked by acidic pH change (Figure 6B, red trace), which may be attributed to the aggregate of a decreased affinity of EGTA as well as the dye for Ca^2+^ in acidic pH (Lattanzio, 1990). This is followed by a modest Ca^2+^ uptake (Figure 6B, red trace), during which the membrane potential gradually depolarizes (Figure 7, pink trace, from 840 to 1200 s). As the membrane potential settles to a new depolarized value, there is no more uptake and the extra-matrix Ca^2+^ attains a steady-state. Alkaline buffer pH (between 7.4 and 7.5 pH units), on the other hand, did not have an effect, which may be attributed to the fact that the extra-matrix pH was similar to previously reported values of matrix pH (∼ 7.6) (Poburko et al., 2011), so that there is a negligible pH gradient across the IMM. On the other hand, the increase in Ca^2+^ efflux and membrane depolarization in acidic buffer pH is consistent with other reports that CHE activity is powered by the dissipation of cross-matrix pH gradient generated by the electron transport chain (Brand, 1985; Tsai et al., 2014; Shao et al., 2016; Haumann et al., 2019). It suggests the possibility that CHE activity may not be electroneutral, as is generally accepted and has been suggested by previous studies (Pozzan et al., 1977; Brand, 1985; Tsai et al., 2014). However, it is also possible that the depolarization induced by acidic buffer pH may involve other pH-dependent mechanisms that have not been the focus of this study and need to be explored in future studies. Nevertheless, the finding that acidic extra-matrix pH increases the rate of Ca^2+^ efflux via the CHE, indicates that under ischemic conditions, cardiac mitochondrial CHE may play a role in Ca^2+^ efflux to avoid mitochondrial Ca^2+^ overloading (Haumann et al., 2010). The reported higher expression of the CHE protein in cardiac mitochondria compared to hepatic mitochondria may reflect a reserve capacity under pathophysiological conditions.

## Conclusion

The current study shows that the CHE activity is stimulated in response to two triggers: (1) a pH gradient across the IMM and (2) changes in matrix Ca^2+^, including matrix free Ca^2+^ and Ca^2+^ buffering. The results point to a previously unattributed unique involvement of CHE with the Ca^2+^ handling capacity of mitochondria. Understanding how CHE activity is associated with mitochondrial Ca^2+^ handling will have implications for pathophysiological conditions such as ischemia/reperfusion injury.

## Data Availability Statement

All datasets generated for this study are included in the article/Supplementary Material.

## Ethics Statement

The animal study was reviewed and approved by the Medical College of Wisconsin Institutional Animal Care and Use Committee.

## Author Contributions

W-MK, AC, and LG designed the initial study. LG, GN, and JM performed the experiments, analyzed the data, and prepared the figures. LG, GN, JM, DS, AC, and W-MK were involved in critical interpretation and discussion of the results, and wrote and edited the manuscript. All authors have read and approved the final version of the manuscript.

## Conflict of Interest

The authors declare that the research was conducted in the absence of any commercial or financial relationships that could be construed as a potential conflict of interest.
