# Supplementary material for: Total Matrix Ca2+ Modulates Ca2+ Efflux via the Ca2+/H+ Exchanger in Cardiac Mitochondria
Source: Front Physiol. 2020 Sep 16;11:510600. doi: 10.3389/fphys.2020.510600 (PMC7526510; doi:10.3389/fphys.2020.510600)
Supplement: Supplementary file 1 [file Data_Sheet_1.pdf]

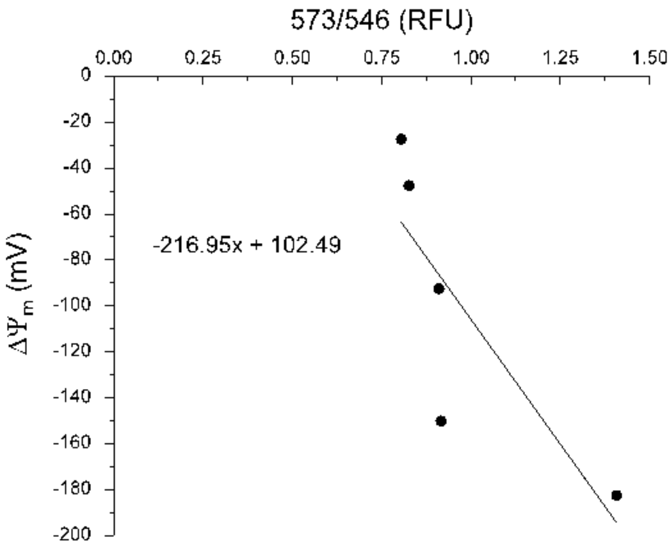

Fig S1 Calibration curve for TMRM derived as described in Methods

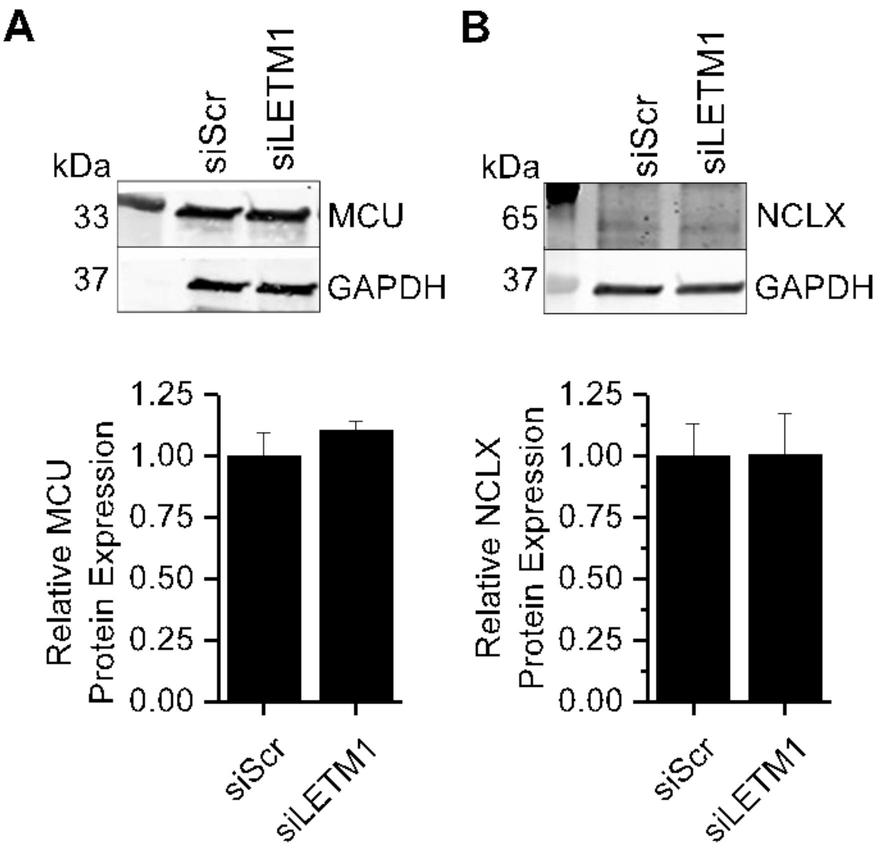

Figure S2. The protein expression of MCU (A) and NCLX (B) determined in H9c2 cells treated with scrambled (siScr) and LETM1 siRNA (siLETM1) by western blotting. GAPDH was used as a loading control. Bars represent mean ± SEM from three independent experiments.
